# The 10% Rule in Sentinel Node Biopsy: Is Removal of All Threshold Nodes Necessary?

**DOI:** 10.1002/ohn.1323

**Published:** 2025-05-29

**Authors:** Jake Langlie, Nicholas DiStefano, Jaylou Velez‐Torres, Carmen Gomez‐Fernandez, Russ A. Kuker, Francisco J. Civantos

**Affiliations:** ^1^ Department of Otolaryngology University of Miami Miller School of Medicine, Division of Head and Neck Surgery Miami Florida USA; ^2^ University of Miami Miller School of Medicine Miami Florida USA; ^3^ Department of Pathology University of Miami Miller School of Medicine, Division of Head and Neck Pathology Miami Florida USA; ^4^ Department of Radiology University of Miami Miller School of Medicine, Division of Nuclear Medicine Miami Florida USA

**Keywords:** gamma probe threshold, head and neck cancer, metastasis, sentinel lymph node biopsy

## Abstract

**Objective:**

Sentinel lymph node biopsy (SLNB) procedures have been guided by the “10% rule,” which stipulates that any lymph node (LN) with gamma probe activity (GPA) measured at 10% of the most radioactive sentinel lymph nodes (SLNs) should be excised. We hypothesized that it would be unusual for a SLN with less than 20% GPA to harbor the only nodal metastasis in a patient.

**Study Design:**

Retrospective analysis.

**Setting:**

Patients with head and neck cutaneous and oral malignancy who underwent SLNB at a tertiary medical center from 2013 to 2023.

**Methods:**

All patients during this period had each SLN recorded with anatomic location and numerical GPA. SLNs which harbored metastatic disease were categorized by GPA, and, in particular, cases that met the 10% threshold, but were less than 20% of the hottest node, were highlighted.

**Results:**

A total of 93 patients presenting with head and neck malignancy that underwent SLNB were included in the study. In total, 433 SLNs were evaluated. Only 1 of these 93 patients (1.1% [95% CI: 1.0%‐1.2%]) had a solitary positive SLN that was 10% to 20% of the hottest node and represented the only known SLN metastasis in that patient. In total, 36 patients (39% of patients) would have had one or more fewer SLN excisions without a change in their staging.

**Conclusion:**

If seeking adherence to a 10% threshold complicates the procedure excessively or generates an excessive number of SLN excisions, a 20% threshold could be used. Based on our study, the risk of subsequent failure to diagnose nodal metastases would be acceptably low.

Lymph node (LN) metastasis remains one of the most important prognostic indicators when evaluating malignancies of the head and neck (H&N). Many techniques have been utilized to diagnose the spread of cancer within the LNs including radiographic imaging,^1^ fine needle aspiration of suspicious LNs,^2^ and ultrasound.^3^ However, many of these techniques rely on the expertise of the interpreter, have a high rate of false positivity,^3^ result in indeterminate diagnosis,^2^ miss occult malignancy in smaller LNs,^1^ and have a low threshold for intervening,^3^ leading to excess surgical intervention.

The term “sentinel lymph node” (SLN) was coined by Gould et al in a case of parotid cancer, to describe the stepwise metastasis of an anatomic area through a “gatekeeping” LN or “sentinel” node.^4^ The sentinel lymph node biopsy (SLNB) procedure was developed based on blue dye initially by Morton,^5^ and later, radiotracer was introduced by Alex and Krag.^6^ This technique has been used in various surgical subspecialties to determine the extent of lymphatic spread of a malignancy in a less invasive manner than an extensive formal lymphadenectomy. The SLNB has been shown to reduce morbidity to the patient, decrease operative times, and can be a therapeutic technique to remove occult malignancy.^7^

The SLNB procedure today involves injecting a radiotracer with or without dye into the primary site and having the radiotracer or dye drain to the lymphatic basin. After waiting for the tracer to distribute through the lymphatic basin, the surgeon can find the “hottest” node indicating that which concentrates the highest gamma radioactivity. Using a probe that measures gamma radioactivity, surgeons can sequentially remove nodes with high gamma probe activity (GPA), usually in order of greatest uptake. Subsequent histopathologic analysis determines whether there are subclinical metastases in the LNs.^8^ The “hottest nodes” are usually the first echelon node—defined as “sentinel nodes”—and exhibit the highest GPA.^9^

SLNB procedures have been used for H&N cutaneous and mucosal malignancy as an alternative to performing an elective neck dissection (END) on selected patients.^10^, ^11^ In oral cavity cancer, a negative SLN has been shown in pathologic validation trials to have a 95% negative predictive value regarding the status of the remainder of the lymphatic basin.^12^ Traditionally, if a positive SLN is encountered, the patient is returned to surgery for completion of lymphadenectomy, though other treatment options including radiation therapy and immunotherapy are sometimes utilized. The SLNB procedure can potentially mitigate multiple morbidities of an END including lymphedema,^13^ shoulder dysfunction,^14^ and excess pain.^15^ The technology is a tool, guiding the surgeon to perform a targeted excision of occult metastasis. The technology has been utilized in various malignancies of the H&N including squamous cell carcinoma,^10^ melanoma, and other cutaneous malignancies.^16^

To build a measure of safety into the SLNB procedure, the 10% rule has traditionally been implemented.^17^ The “10% rule” stipulates that any SLN with GPA measured at 10% of the hottest nodes should also be considered a SLN, and therefore, excised.^18^, ^19^ Support for the 10% rule came from the Sunbelt melanoma trial, where higher thresholds would have led to higher false negative rates.^20^ This trial was multi‐institutional, involving multiple authors and techniques, and was a series of patients who all had melanoma. The 10% rule has been maintained as we introduce the SLNB technique in other diseases and histopathologies. However, the 10% rule has recently been under scrutiny, specifically in melanoma excision, where recent studies have demonstrated success removing less nodes without changing the staging of the patient.^19^

The objective of our study is to investigate whether we can simplify SLNB procedures by allowing flexibility in the threshold GPA between 10% and 20% of the hottest node in malignancies localized to the H&N anatomic region. Although the 10% threshold could be maintained as the initial plan, surgeons could use a 20% threshold if the lower activity nodes are difficult to access. Based on our experience, we hypothesized that, with careful attention to proper injection technique, it would be unusual for an SLN with less than 20% GPA to harbor a metastasis. This study was designed to determine if rigid application of the 10% rule is necessary, and if SLNB could be simplified by introducing flexibility in the threshold GPA, with an option for a higher threshold for surgical excision of the SLN.

## Methods

The following study was approved by the University of Miami's Human Subjects Research Office Institutional Review Board, IRB#: 20110664. Patient records were retrieved at our tertiary academic institution from 2013 to 2023 for patients presenting with H&N cutaneous and oral malignancy who underwent SLNB by the senior author. The radiocolloid used by our service during this period was technetium (^99m^Tc) tilmanocept. Nuclear imaging of SLN included initial planar imaging for 15 to 30 minutes, followed by single‐photon emission computed tomography (SPECT)‐computed tomography (CT) fused images. Injections for nuclear mapping were performed in all cases by the senior surgeon himself, with significant attention to evenly distributing radiocolloid around the lesion with superficial peripheral injections, and occasionally a deeper injection for thicker tumors. We did not inject blue dye as an additional marker during this period of time.

Patient demographics (age and gender), number of excised nodes, histopathological diagnosis, node positivity rate, pathological TNM staging (postoperative), clinical staging, and gamma probe uptake were collected from the electronic health record. More specifically, node count was collected from the intraoperative surgical pathology record. It should be noted that the senior surgeon was scrubbed and directed the surgery for all of our cases. All patients during this period had each node recorded with anatomic location and numerical GPA. Gamma probe uptake percentage was calculated by dividing the gamma probe reading from the selected LN by that of the “hottest” SLN in the lymphatic drainage basin. Patients were evaluated for detection of metastatic disease through permanent histopathology of SLN removed during neck or parotid exploration that met the “10% threshold rule.”

## Results

### Overview

A total of 93 patients presenting with either H&N cutaneous malignancy or oral cavity malignancy that underwent SLNB from 2013 to 2023 were included in the study. In total, 40% of the patients included in the study were female, and the median age within the study was 64.2 ± 15.7 years (Table 1). In total, 433 SLNs were evaluated. Histologic patient diagnoses included mucosal squamous cell carcinoma (n = 37, 39.8%), melanoma (n = 33, 35.4%), cutaneous squamous cell carcinoma (cSCC; n = 10, 10.8%), Merkel cell carcinoma (MCC; n = 9, 9.7%), and other rare cutaneous malignancies (n = 4, 4.3%). Only 1 of these 93 patients (1.1% [95% CI: 1.0%‐1.2%]) had a solitary positive SLN that was 10% to 20% of the hottest node and represented the only known SLN metastasis in that patient. Two patients with melanoma had positive SLNs that were 9% and 15% of the hottest nodes, but both had foci of melanoma in nodes with higher GPA. None of the patients with histology other than melanoma had a positive node with GPA less than 20%.

**Table 1:** Patient Demographics

	Mucosal squamous cell carcinoma (n = 37)	Melanoma (n = 33)	Cutaneous squamous cell carcinoma (n = 10)	Merkel cell carcinoma (n = 9)	Rare malignancies^a^ (n = 4)	Total
Gender						
Male	21 (57%)	20 (61%)	9 (90%)	4 (44%)	2 (50%; AS)	56 (60%)
Female	16 (43%)	13 (39%)	1 (10%)	5 (56%)	2 (50%; PTC/SeCC)	37 (40%)
Age						
20‐30	1 (3%)	1 (3%)	1 (10%)	‐	‐	3 (3%)
31‐40	1 (3%)	2 (6%)	1 (10%)	‐	‐	4 (4%)
41‐50	8 (21%)	3 (10%)	‐	‐	‐	11 (12%)
51‐60	10 (27%)	3 (10%)	‐	1 (11%)	1 (25%, SeCC)	15 (16%)
61‐70	5 (14%)	11 (33%)	3 (30%)	1 (11%)	‐	20 (22%)
71‐80	8 (21%)	7 (20%)	3 (30%)	5 (56%)	3 (75%, SeCC, AS, PTC)	26 (28%)
81‐90	4 (11%)	6 (18%)	2 (20%)	2 (22%)	‐	14 (15%)
Average (SD^b^)	60.4 (15.0)	64.5 (16.3)	65.5 (19.3)	74.9 (8.0)	69.5 (10.5)	64.2 (15.7)
Primary subsite						
Auricle	‐	9 (27%)	5 (50%)	‐	‐	14 (15%)
Cheek	‐	8 (24%)	1 (10%)	4 (45%)	‐	13 (14%)
Floor of mouth	2 (5%)	‐	‐		‐	2 (2%)
Glabella	‐	‐	‐	1 (11%)	‐	1 (1%)
Hard palate	1 (3%)	‐	‐	‐	‐	1 (1%)
Lower alveolar ridge	2 (5%)	‐	‐	‐	‐	2 (2%)
Lower eyelid	‐	1 (3%)	‐	‐	‐	1 (1%)
Lower lip	4 (11%)	1 (3%)	‐	‐	‐	5 (5%)
Neck	‐	3 (10%)	‐	‐	‐	3 (3%)
Nose	‐	1 (3%)	3 (30%)	2 (22%)	‐	6 (6%)
Oral tongue	23 (62%)			1 (11%)	1 (25%, PTC)	25 (27%)
Retromolar trigone	1 (3%)	‐	‐	‐		1 (1%)
Scalp	‐	10 (30%)	1 (10%)	‐	1 (25%, AS)	12 (13%)
Upper eyelid	‐	‐	‐	‐	2 (50%, SeCC)	2 (2%)
Upper lip	4 (11%)	‐	‐	1 (11%)	‐	5 (5%)

^a^ Sebaceous cell carcinoma (n = 2, SeCC), angiosarcoma (n = 1, AS), and papillary thyroid carcinoma (PTC).

^b^ SD = standard deviation.

The number of SLNs removed varied by patient with patients having between one and eight excisions performed (mean = 4 with SD = 1.6). In the study, 36 patients (39% of patients) would have had one or more fewer excisions without a change in their staging. Out of the 93 patients in the study, 59 fewer SLN excisions could have been performed without altering the clinical course of the study participants. Only one patient with a T2a, N1a melanoma violated this rule in the study with only one positive SLN that was 10.4% of the hottest sentinel node. The GPAs of all SLNs that harbored malignancy were also recorded (N = 21).

### Gamma Probe Uptake: Melanoma

In total, 33 patients in the study had a histopathological diagnosis of melanoma at the primary site. Of the 33 patients, 9 had a coinciding positive histopathologic diagnosis of melanoma in a SLN. Two patients had positive SLNs that were 9% and 15% of the hottest nodes, but both had foci of melanoma in nodes with higher GPA. One patient (1.1% of all patients, 3.3% of melanoma patients) had a solitary positive SLN that was 10% to 20% of the hottest node (10%) and represented the only SLN metastasis identified.

**Figure 1:**
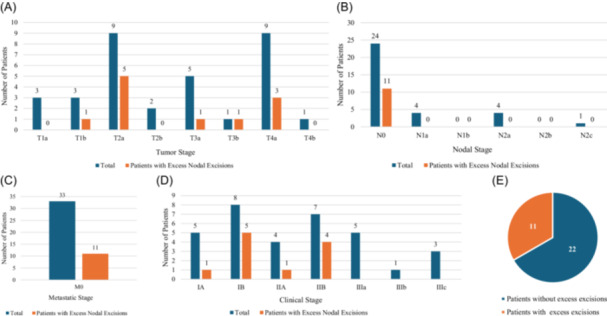
Patients with melanoma and patients with excess nodal excision. (A) Tumor staging, (B) nodal staging, (C) metastatic stage, (D) clinical stage, and (E) excess nodal excisions.

Tumor stage did not appear to be correlated with the rate of “excess” SLN excisions (between 10% and 20% of the hottest node) in patients with melanoma with 0% of T1a tumors (n = 0/3 patients), 33% of T1b tumors (n = 1/3 patients), 56% of T2a tumors (n = 5/9 patients), 0% of T2b tumors (n = 0/2 patients), 20% of T3a tumors (n = 1/5 patients), 100% of T3b tumors (n = 1/1 patients), 33% of T3a tumors (n = 3/9 patients), and 0% of T4b tumors (n = 0/1 patients) demonstrating “excess” SLN dissections without yielding additional metastatic nodes (Figure 1A). Coincidentally, all patients with positive SLNs did not have any nodes removed falling into the “excess” 10% to 20% range, and all patients who had nodes radioactively tagged that fell into the 10% to 20% (“excess”) range, were N0 when final pathology became available. The group with SLNs marked and removed in the 10% to 20% range represented 46% (11/24) of melanoma patients with N0 disease and represented 33% of all patients with melanoma in the study (11/33) (Figure 1B). All patients with excess nodal excisions (n = 11) between 10% and 20% of the hottest GPA threshold had M0 disease (33% of patients) (Figure 1C). Patients with melanoma with “excess” SLN excisions tended to have lower stage disease including stage IA (n = 1/5), stage IB (n = 5/8), stage IIA (n = 1/4), and stage IIB (n = 4/7). No patients with stage IIIa to IIIc disease demonstrated excess SLN excisions per the criteria in our study (Figure 1D). Similarly, no patients with stage IVa or IVb disease demonstrated excess nodal excisions per the criteria in our study. In total, 33% of patients with melanoma had excess SLN excisions according to the recommendations of this study (Figure 1E). These latter results appeared to be coincidental, rather than being related to decision‐making during surgery, or intraoperative findings. Gross disease in the cervical lymphatics was not encountered in our study, probably because patients with clinically involved LNs were identified on preoperative imaging.

### Gamma Probe Uptake: Mucosal Squamous Cell Carcinoma

In total, 37 patients had a primary diagnosis of mucosal squamous cell carcinoma and underwent SLNB. Of those 37 patients, 8 patients had a coinciding positive histopathologic diagnosis of squamous cell carcinoma in a SLN. Of these eight cases, three had nodules that were positive on histopathology that had less GPA than the hottest node. This included one case of SCC of the tongue that had multiple positive LNs in level 2 (GPA = 5389) and 3 (GPA = 22,004, hottest node), demonstrating the lowest percentage of the hottest SLN among SCC cases at 24%. Two cases, also both oral cavity SCC of the tongue, only had one positive SLN that were 32% and 35% of the hottest node, respectively.

**Figure 2:**
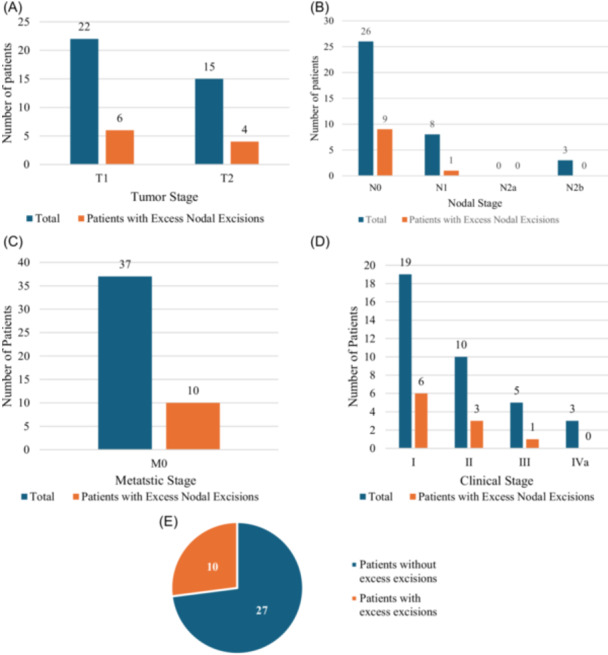
Patients with mucosal squamous cell carcinoma and patients with excess nodal excision. (A) Tumor staging, (B) nodal staging, (C) metastatic stage, (D) clinical stage, and (E) excess nodal excisions.

All patients with mucosal squamous cell carcinoma that underwent SLNB were T1 (n = 22) and T2 (n = 15) primary site stage, with nearly equal percentage of “excess” SLNs excised per group: T1 at 27% with 6/22 patients and T2 at 27% with 4/15 patients (Figure 2A). Patients with mucosal squamous cell carcinoma that had excess LN dissection were either N0 (n = 9/26, 35%) or N1 (n = 1/8, 13%). No patients with nodal stage N2b had “excess” LNs removed (Figure 2B). All patients with “excess” SLNs removed had M0 disease (n = 10/37, 27%) (Figure 2C). Patients with excess SLNs removed tended to have lower clinical stage including 32% of patients with stage I disease (n = 6/19), 30% of patients with stage II disease (n = 3/10), 20% of patients with stage III disease (n = 1/5), and 0% of patients with stage IVa disease (Figure 2D). In total, 27% of patients with mucosal SCC had excess nodal excisions (between 10% and 20% of the hottest node) as defined for this study (Figure 2E).

### Gamma Probe Uptake: Cutaneous Squamous Cell Carcinoma and Merkel Cell Carcinoma

In total, 10 patients had a primary diagnosis of cutaenous squmous cell carcinoma (cSCC) and underwent SLNB. Of those 10 patients, 1 patient had a coinciding positive histopathologic diagnosis of squamous cell carcinoma in an SLN. The one case corresponded to a skin cancer that had metastasized to the parotid had one positive node that was 68% of the hottest SLN in level II.

Nine patients in the study had a diagnosis of Merkel cell carcinoma (MCC). Three patients had a coinciding positive histopathologic diagnosis of MCC of the lymphatics on SN biopsy. Of these, only one case had a positive SLN that had a 32% GPA of the hottest SLN that also had a positive histopathologic diagnosis.

**Figure 3:**
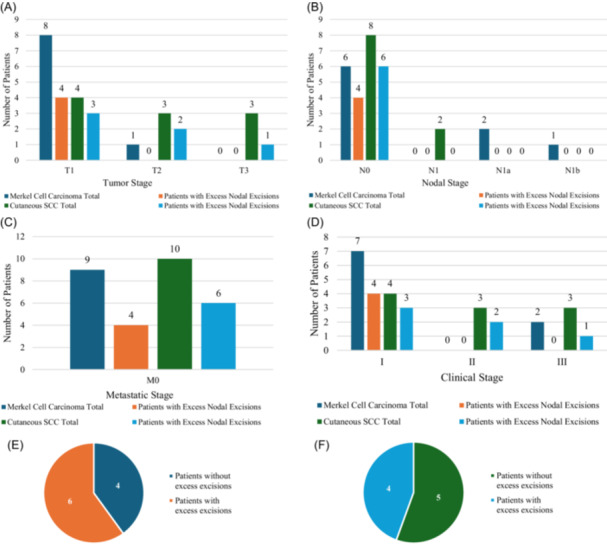
Patients with Merkel cell carcinoma (MCC) and cutaneous squamous cell carcinoma (cSCC). (A) Tumor staging, (B) nodal staging, (C) metastatic stage, (D) clinical stage, and excess nodal excisions for (E) MCC and (F) cSCC.

The majority of patients with MCC were T1 (n = 8) with 50% of those patients having an “excess” SLN excision (n = 4/8). One patient with MCC had stage II disease (Figure 3A). This may be due to a tendency for clinically positive nodes in more advanced stage patients, and a policy of going directly to complete lymphadenectomy with advanced primary site disease. Patients with cSCC with “excess” nodal excisions tended to have lower primary site stage including 75% of patients with T1 disease (n = 3/4), 66% of patients with T2 disease (n = 2/3), and 33% of patients with T3 disease (n = 1/3) (Figure 3A). All patients with MCC or cSCC with “excess” nodal excisions were N0—66% of MCC patients (n = 4/6) and 75% of cSCC patients (n = 6/8) (Figure 3B). All patients with MCC or cSCC with excess nodal excisions did not have distant metastasis—44% of MCC patients with M0 disease (n = 4/9) and 60% of cSCC patients (n = 6/10) had excess incisions (Figure 3C). All patients with MCC with excess nodal excisions were clinical stage I (57%, n = 4/7 patients with stage I MCC) (Figure 3D). Patients with cSCC with excess nodal excisions tended to have lower clinical stage including 75% of stage I cSCC (n = 3/4), 66% of stage II (n = 2/3), and 33% of stage III (n = 1/3) (Figure 3D). In total, 60% of patients with MCC in the study had “excess” nodal excisions (Figure 3E). In total, 44% of patients with cSCC had “excess” SLN excisions (Figure 3F).

## Discussion

The SLNB technique is based on an accurate injection encompassing the field of the primary tumor and uptake of the tracer into the lymphatics. There is always some unevenness to the injection, which can represent a limitation to the procedure, and there can also be some variability in uptake of the radiotracer to the lymphatics. In addition, for a larger tumor, different parts of the lesion can drain in different directions. For these, as well as possibly other reasons that are not completely understood, it has been shown that the hottest node is not always the positive node.^21^ Therefore, the 10% rule was created to safeguard against missing occult malignancy. However, our study along with the other recent studies continues to put the rule under scrutiny.^18^, ^19^, ^22^

One of 93 patients (1.1% [95% CI: 1.0%‐1.2%]) presented with a single positive SLN between 10% and 20% of the most radioactive SN. The primary tumor in this case was melanoma. Two additional patients with melanoma had positive SNs that were 9% and 15% of the hottest nodes. However, both of these patients had foci of melanoma in nodes with higher GPA. Therefore, the pathological status of the neck lymphatic basin (positive vs negative) would not have changed in either of these later cases. All three of these metastases that occurred in less radioactive SLN only occurred in patients with melanoma, a finding of unclear importance.

In our study, 36 patients (39% of patients) would have had one or more fewer SLN excisions without a change in their staging. Out of the 93 patients in the study, 59 fewer excisions could have been performed without altering the clinical course of the study participants. Only one patient with a T2a, N1a melanoma violated this rule in the study with only one positive SLN that was 10.4% of the hottest node. On final pathology, the rate of having cancer in an SLN that is between 10% and 20% of the hottest node would be approximately 1.1% based on our study. We believe that this study aids the practitioner, as they can selectively implement fewer SLN excisions for their patients, when clinically appropriate, without having stage migration or missing regional LNs with micrometastasis.

Our results are different from those of Kroon et al^18^ in a series of 561 melanomas, who found that increasing the threshold to 20% led to a 4% increase in false negative cases, as opposed to our finding of a 1.1% increase in false negatives. Though numerically close, the difference led to very different conclusions, as Kroon et al advocated for maintaining the 10% threshold. This was despite the fact that they calculated for their institution greater than $7000 in savings per patient by increasing the threshold to 20%. The two studies are different, in that ours had a mix of histopathologic diagnoses, and focused on the SLNB technique itself. We used Technetium (^99m^Tc) tilmanocept as our radionuclide, whereas the prior study used ^99m^Tc‐sulfur colloid. In addition, our study was restricted to the H&N region alone. All of our surgeries were performed by a single senior surgeon, and injections were all performed by the surgeon himself. We conjecture that a better injection technique could lead to less need to sample additional nodes with lower GPA.

In contrast, Murphy et al^19^ found that increasing the threshold would have led to no additional false negatives in 665 patients with melanoma and reached conclusions similar to ours: that the threshold could be carefully raised, particularly if the additional nodes were in the same lymphatic basin as “hotter” nodes. Their study used ^99m^Tc‐human serum albumin colloid as the radionuclide, which is not available in the United States. They advocated for “hottest two and blue” as a new rule, which takes the increase in threshold much higher than just 20%. Both of these prior studies were focused on melanoma, whereas we have included a variety of H&N histopathologies.

The most recent publication that looks at the “10% rule” by Tellman et al^23^ was focused on cancer of the oral cavity. Using either tilmanocept or nanocolloid as the radiotracer, this group evaluated the 10% rule for 66 T1/T2 oral cavity cancers. Only one of their patients had the status of one side of the neck changed by an SLN that was less than 20% and more than 10% of the hottest node. Thus, the neck on one side was upstaged 1.4% of the time by applying the 10% rule, a number almost the same as that produced in our own study. They reached a different conclusion, however, as the authors expressed concern that an additional five nodes with cancer, in the necks where a hotter SLN had resulted positive, would have been left behind. Therefore, they argued for maintaining the 10% rule.

We also argue for maintaining the 10% rule when it is easy to comply with. However, with such a low incidence of conversion from N0 to positive, we would argue that surgeons, as a minimum, should understand the numbers involved and have flexibility in their practice. Some cases where we foresee the implementation of this flexibility include cases where a lower GPA SLN happens to be deep in the parotid, in the parapharyngeal space, under the clavicle, or is otherwise difficult to remove. If the same node was over 20%, dissection and removal would be extremely important. Our argument, it should be noted, is predicated on the concept that a side of the neck that is deemed positive will receive more treatment, most often with completion neck dissection, particularly for oral cavity cancer. For melanoma, under current protocols, immunotherapy might be given first.

To summarize, in our own study, for H&N malignancies of heterogeneous histopathology, in terms of the surgical technique, we found that removal of SLN with radioactivity between 10% and 20% of the “hottest” node generated few malignant nodes.^18^, ^19^ Excision of these SLN changed the status of the lymphatic basin in one patient out of 93 patients in the cohort (1.1%). Another recent trial with 66 oral cavity cancers resulted in a similar number of 1.4%.^23^ A large trial of more than 600 patients with melanoma found no case where a node between 10% and 20% changed the status of the lymphatic basin.^19^ One other study had a higher conversion rate to positivity at 4% in 561 melanomas.^18^ Based on all of these data, with rates of conversion to positivity of the lymphatic basin at less than 5%, we make the argument that at a minimum, one could consider a flexible threshold between 10% and 20% of the SN with the greatest GPA, particularly if the borderline LN in question is difficult to excise. More specifically, if seeking adherence to a 10% threshold—relative to a less stringent 20% threshold—requires dissection of nerves that have not yet been identified, opening of new lymphatic basins with additional incisions, or if the number of SLN excised is already greater than four or five LN, a 20% threshold could be utilized. This adjustment makes the procedure more practical as a minimally invasive intervention.

It is important to acknowledge that we selected a group based on the SLNB technique and the anatomic region of the H&N but had heterogeneity in terms of histopathology. This can create heterogeneity in the data. However, we felt it was important to pool H&N cases specifically, as the H&N has been generally considered more technically difficult, and one might postulate that this would be a group where additional LN sampling might be important—an assumption that our study did not support. Until the publication from Tellman et al,^23^ there had not been prior studies looking at “excess” SLN excision for mucosal squamous cell carcinomas, and there still is no study that looks at the same question in cSCC or MCCs, so that this is a first attempt to include these additional histopathologies. The data presented in this study is stratified by pathology to allow the practitioner to independently evaluate and apply our findings to their own clinical practice. However, larger numbers of patients would be needed to answer critical questions regarding the 10% rule for these groups. Some of these critical questions include the following:

1. Does the type of primary cancer (melanoma, mucosal squamous cell carcinoma, cSCC, or MCC) affect the drainage pattern and the 10% rule?
2. Does the drainage pattern of HN subsites versus other sites (extremity, trunk) affect the drainage pattern and the 10% rule?
3. Does the stage of the primary site affect the drainage patterns and the 10% rule?

One can conjecture that these factors might have an effect on lymphatic drainage and on the “10% rule.” In fact, it has been our bias that squamous cell carcinoma is more likely to have gross involvement in a small number of nodes, whereas melanoma and Merkel cell are more likely to have multiple micrometastases without macrometastases (relative to squamous cell carcinoma). However, we have not proven this here, and more studies with a larger number of patients in each of these subgroups would be needed to answer these questions.

## Conclusions

We have compared the “10% rule” for SLN excision to a hypothetical “20% rule.” The “10% rule” generated a greater number of SLN excisions with minimal incremental identification of metastases. For a procedure that is designed to be minimally invasive relative to formal lymphadenectomy, this represents a conceptual problem that could be revisited. We would suggest consideration of a flexible threshold between 10% and 20% of the “hottest” SN. If seeking adherence to a 10% threshold complicates the procedure excessively, or generates an excessive number of nodal excisions, the 20% threshold could be used. In this study, the excision of SLN with GPA less than 20% of the “hottest” node converted the lymphatic basin to positive for malignancy in only 1.1% of cases.

## Author Contributions

**Jake Langlie**, conception, design, acquisition of data, analysis, draft, and approval of manuscript; **Nicholas DiStefano**, conception, acquisition of data, analysis, draft, and approval of manuscript; **Jaylou Velez‐Torres**, conception, revising, and approval of manuscript; **Carmen Gomez‐Fernandez**, conception, revising, and approval of manuscript; **Russ A. Kuker**, conception, revising, and approval of manuscript; **Francisco J. Civantos**, conception, design, analysis, revising, and approval of manuscript.

## Disclosures

### Competing interests

The authors declare no conflict of interest.

### Funding source

None.
